# BRCA1 and NORE1A Form a Her2/Ras Regulated Tumor Suppressor Complex Modulating Senescence

**DOI:** 10.3390/cancers15164133

**Published:** 2023-08-16

**Authors:** Nicholas Nelson, Raphael Jigo, Geoffrey J. Clark

**Affiliations:** 1 Department of Chemistry, US Naval Academy, Annapolis, MD 21402, USA; 2 Department of Pharmacology and Toxicology, University of Louisville, Louisville, KY 40202, USA

**Keywords:** RAS, BRCA1, Her2, senescence, NORE1A

## Abstract

**Simple Summary:**

The loss of function of the BRCA1 tumor suppressor is common in breast cancer, but experimentally, the BRCA1 inactivation promotes cell senescence. The overstimulation of RAS signaling via Her2 upregulation is also common in breast cancer, yet it can also lead to senescence. A significant percent of primary breast tumors exhibit both defects. So how do they overcome the senescence response to become successful tumors? Here we show that the RAS senescence effector NORE1A can complex with BRCA1 and that loss of NORE1A abrogates the senescence-inducing effects of Her2 and BRCA1 dysregulation. Database analysis shows that NORE1A loss of expression is common in primary breast tumors and correlates with BRCA1 loss in Her2+ but not Her2− cases.

**Abstract:**

BRCA1 is a tumor suppressor with a complex mode of action. Hereditary mutations in BRCA1 predispose carriers to breast cancer, and spontaneous breast cancers often exhibit defects in BRCA1 expression. However, haploinsufficiency or suppression of BRCA1 expression leads to defects in DNA repair, which can induce DNA damage responses, leading to senescence. Activating mutation or overexpression of the Her2 oncoprotein are also frequent drivers of breast cancer. Yet, over-activation of Her2, working through the RAS oncoprotein, can also induce senescence. It is thought that additional defects in the p53 and Rb tumor suppressor machinery must occur in such tumors to allow an escape from senescence, thus permitting tumor development. Although BRCA1 mutant breast cancers are usually Her2 negative, a significant percentage of Her2 positive tumors also lose their expression of BRCA1. Such Her2+/BRCA1− tumors might be expected to have a particularly high senescence barrier to overcome. An important RAS senescence effector is the protein NORE1A, which can modulate both p53 and Rb. It is an essential senescence effector of the RAS oncoprotein, and it is often downregulated in breast tumors by promotor methylation. Here we show that NORE1A forms a Her2/RAS regulated, endogenous complex with BRCA1 at sites of replication fork arrest. Suppression of NORE1A blocks senescence induction caused by BRCA1 inactivation and Her2 activation. Thus, NORE1A forms a tumor suppressor complex with BRCA1. Its frequent epigenetic inactivation may facilitate the transformation of Her2+/BRCA1− mediated breast cancer by suppressing senescence.

## 1. Introduction

The disruption of the function of the BRCA1 tumor suppressor plays a key role in the development of many breast cancers. Individuals carrying hereditary mutations in BRCA1 have an elevated risk of developing breast cancer [1], and many breast tumors exhibit somatic mutations or a reduced expression of BRCA1. This can involve point mutations, promoter methylation, or allelic loss [2,3,4,5].

BRCA1 has multiple functions, which has made fully understanding its biology difficult [6]. It is involved in the regulation of apoptosis [7], mitophagy [8], centrosomes [9], transcriptional activity [10], chromatin remodeling [11], and senescence [12]. However, it is perhaps best known for its role in maintaining genomic stability [13]. It plays a key role in the regulation of DNA damage checkpoints that arrest the cell to allow time for DNA repair [14]. It also has a vital role in coordinating the DNA repair processes required for the homologous recombination repair of double-strand breaks caused by DNA-damaging agents or the collapse of stalled replication forks [15,16,17,18]. Thus, defects in the BRCA1 function led to genetic instability, predisposing to the development of cancer.

BRCA1 has also been shown to participate in the regulation of senescence. Excess BRCA1 activity can promote senescence [19], in part via p53. This makes perfect sense, bearing in mind the well-characterized role of BRCA1 in cell cycle checkpoint control [20]. However, the loss of BRCA1 can also induce the senescence phenotype [21]. Senescence promoted by BRCA1 loss, appears to involve the Rb protein and has been described as haploinsufficiency-induced senescence (HIS) [22].

In addition to BRCA1 inactivation-mediated senescence (HIS), senescence may also be induced by the aberrant activation of oncoproteins. This is known as oncogene-induced senescence or OIS [23]. The classic mediator of OIS is the RAS oncoprotein [24,25]. The RAS oncogene is frequently mutated in many types of cancer and plays a key role in the initiation and development of malignancy [26]. Although less than 2% of primary breast tumors contain activating Ras mutations [27,28], the Her2 oncoprotein signals through Ras [27,29], and Her2 mutation or amplification occur in ~25–30% of all breast cancers [30,31]. Excessive activity of Her2 (which activates RAS via the GRB2/SOS exchange factor system RAS [32,33]) can also induce senescence [34,35], involving the activation of the p53/p21CIP1 pathway [36] and possibly the Rb pathway [37]. Both of these senescence effects, OIS and HIS, may serve as an evolved defense mechanism against cancer development that must be overcome in order for successful transformation [22,34].

Her2 overexpression is not common in hereditary BRCA1 tumors, and the majority of findings suggest either no association or an inverse correlation between Her2 overexpression and BRCA1 mutation in primary breast tumors. However, several reports have shown that BRCA1 expression loss in Her2-positive sporadic breast tumors is associated with worse prognostic features (e.g., higher histological grade, increased proliferation), early progression [38], and a poor survival rate [39,40]. Moreover, one of these studies found that 90% of the Her2-positive tumors in their experimental cohort were, in fact, negative for BRCA1 expression [40].

Yet, if both aberrant Her2 activation induces senescence [12] and BRCA1 haploinsufficiency induces senescence [22], how do Her2+/BRCA1− cells develop into successful tumors without succumbing to senescence? Presumably, some key component of the senescence induction machinery must be subverted to facilitate tumorigenesis.

NORE1A (RASSF5) is a RAS effector protein that directly binds to RAS when RAS is in the active, GTP-bound form [41]. It is a member of the RASSF family of tumor suppressors, which act as scaffolding molecules for the assembly of a variety of signaling complexes involved in apoptosis, DNA repair, and senescence [42]. It allows RAS to modulate the activity of both p53 (via HIPK2) and Rb (via PP1a) to promote senescence [43,44]. The activation of Her2 causes the receptor to assemble a GRB-2/SOS exchange factor complex that activates RAS [32]. Thus, Her2 may be able to stimulate RAS/NORE1A senescence pathways.

NORE1A is inactivated in ~40% of breast cancer cell lines and primary tumors by promoter hypermethylation [45]. Moreover, our database analysis (Breast Cancer Gene-Expression Miner v3.2) [46] indicates that there is a statistically significant relationship (*p* < 0.05, r = −0.27) between reduced expression of BRCA1 and reduced expression of NORE1A in Her2+, but not Her− breast cancers. Consequently, we wondered if the loss of the NORE1A senescence effector might play a role in shifting the balance of proliferation/senescence in Her2+/BRCA1− cells.

We have found that NORE1A forms a Ras/Her2-regulated endogenous complex with BRCA1 and that it co-localizes with BRCA1 at stalled replication forks. The suppression of NORE1A and BRCA1 has a cooperative effect on breast cell transformation. Furthermore, NORE1A inhibition suppresses senescence in Her2+/BRCA1− cells and BRCA1 knockdown suppresses NORE1A-induced senescence. Thus, NORE1A forms a tumor suppressor complex with BRCA1 and may play an important role in connecting Her2 to BRCA1 regulation to modulate the senescence response to oncogenic lesions in breast cancer.

## 2. Materials and Methods

### 2.1. Plasmids and DNA

Full-length human NORE1A plasmids have been described previously [43]. NORE1A-INT domain was generated by cloning AA 170–272 of the full-length NORE1A cDNA as a BglII-EcoRI fragment into a pCDNA vector with an HA epitope tag. All PCR constructs were confirmed by sequencing prior to use. shRNAs for human NORE1A (described in [43]) were obtained from Open Biosystems (Hunstville, AL, USA). GFP-BRCA1 was a gift from Dr. Ray White (Huntsman Cancer Institute, Salt Lake City, UT, USA). shRNAs for human BRCA1 were obtained from Origene (SKU: TG314440), and stable transfected cell lines were obtained after selection with puromycin dihydrochloride (1 μg/mL; Sigma-Aldrich, St. Louis, MO, USA). HER2 CA (V659E) was a gift from Mien-Chie Hung (Addgene [47], and stable transfected cell lines were obtained after selection with G418 Sulfate (300 μg/mL; Invivogen, San Diego, CA, USA).

### 2.2. Tissue Culture and Cell Lines

HEK-293T, MCF-7, and MCF-10A cells were obtained from the ATCC (Manassas, VA, USA). HEK-293T and MCF-7 cells were grown in DMEM (Thermo Fisher, Waltham, MA, USA) with 10% FBS (Valley Biomedical, Winchester, VA, USA) and 1% penicillin/streptomycin (Corning, Corning, NY, USA). MCF-10A cells were grown in DMEM/F12 medium supplemented with 5% horse serum (Gibco, Waltham, MA, USA), 1% penicillin/streptomycin, 0.5 mg/mL hydrocortisone (Sigma-Aldrich), 20 ng/mL human epidermal growth factor (EGF) (Sigma-Aldrich), and 10 μg/mL human insulin solution (Sigma-Aldrich).

Transfections: Stable transfectants were generated by transfecting cells each with 2 μg of plasmid DNA using jetPRIME (PolyPlus, Vectura, France) transfection reagent, as described in the manufacturer’s protocol.

Growth curves: MCF-10A standard growth curves were performed by plating 2 × 10^4^ cells per 60 mm dish (Cellstar, Waltham, MA, USA), and cells from each set were trypsinized (0.25% Trypsin; Corning) and counted manually at the same time each day for four days.

Soft agar assays: Soft agar assays were performed by resuspending cells in standard soft agar mix (0.5 mL penicillin/streptomycin (Corning), 1.8 mL 10xPBS, 1.6 mL serum (Gibco), 30 mL DMEM/F12 medium (Corning), 16 mL melted agar (ThermoFisher), and plating them on a pre-hardened 0.7% agar base in 12-well plates. Anchorage-independent growth was determined by manually counting the number of colonies formed in agar 2 weeks after plating the cells.

Senescence assays: β-galactosidase assays were performed using a BioVison kit (BioVision, Milpitas, CA, USA), as recommended by the manufacturer. Senescent cells were quantified and imaged using an IX50 inverted system microscope with a UPlanFl 4×/0.13 PhL or LCPlanFl 20×/0.40 Ph1 objective (Olympus, Westborough, MA, USA).

Replication stress: To induce replication stress, HEK-293T cells were treated with 2 mM hydroxyurea (Sigma-Aldrich) in cell culture media for 6 h.

Fluorescent microscopy: GFP/YFP and RFP/KATE proteins were visualized at room temperature in live cells in growth medium using an Olympus IX50 inverted microscope with a UPlanFl 100×/1.3 oil immersion objective. Quantification was via imageJ (version 1.52).

### 2.3. Antibodies

Monoclonal and polyclonal NORE1A antibodies have been described previously [43]. Mouse monoclonal HA antibodies were obtained from Thermofisher, and mouse monoclonal GFP and BRCA1 were from Santa Cruz Biotechnology, Santa Cruz, CA, USA Inc. β-actin were obtained from Sigma-Aldrich. Rabbit polyclonal p21CIP and Her2 antibodies were obtained from Cell Signaling Technology, Boston, MA, USA.

### 2.4. Western Analysis and Immunoprecipitation

Cells were lysed in 100 μL RIPA buffer (Sigma-Aldrich) with sodium orthovanadate (Sigma-Aldrich) and protease inhibitor cocktail (Sigma-Aldrich cat # P8340) added just prior to lysis, and the concentration of protein in the lysates was quantified using a Bio-Rad protein assay (Bio-Rad) using a spectrophotometer (abs: 595). Equal amounts of protein lysate were subject to electrophoresis and Western analysis. For co-immune precipitation, cells were lysed in 100–200 μL modified RIPA buffer (150 mM NaCl, 50 mM Tris, pH 7.5, 1% Tergitol NP-40 (Sigma-Aldrich)) with protease inhibitors added prior to lysis as above. Precleared lysates were immunoprecipitated overnight with GFP-Trap agarose beads (Allele Biotech, San Diego, CA, USA) or primary antibody as appropriate and washed 3 × 3 min with lysis buffer, then Western blotted as above.

### 2.5. Image Acquisition and Processing

All images were scanned and quantified using a Pharos FX plus Molecular Imager (Bio-Rad, Hercules, CA, USA) and Quantity One software (Bio-Rad) or ImageJ software (version 1.52). Linear range was determined for individual antibodies by plotting the quantitated densities of Western blotted bands against the initial amount of protein lysate loaded in each lane. To control for variability, the background-subtracted densities of each protein of interest and normalizing control (e.g., beta-actin) were quantified and protein of interest values were divided by relative normalized control values in their respective lanes. For quantitative experiments, each condition was performed in duplicate or triplicate as described. Averages, *p*-values (*t*-test), and fold-changes were calculated, and the results graphed using Excel (Microsoft). Images were compiled using PowerPoint (Microsoft) or Photoshop (Adobe).

## 3. Results

### 3.1. NORE1A Forms a Her2/Ras-Regulated Complex with BRCA1

In breast cell line systems, Her2 induction stimulates OIS (oncogene-induced senescence) [34]; moreover, almost 5% of the cells in Her2+ breast pdx systems stain positive for markers of senescence [48]. Her2 acts through the Ras oncoprotein [29], and Ras can induce senescence via BRCA1 in breast cells, although the exact mechanism remains unclear [49]. We have previously found that the Ras effector NORE1A plays a vital role in connecting Ras to the senescence machinery [50]. Moreover, NORE1A partially localizes to nuclear foci [43], as does BRCA1 [51]. Therefore, we wondered if NORE1A might be acting, in part, via BRCA1.

**Figure 1:**
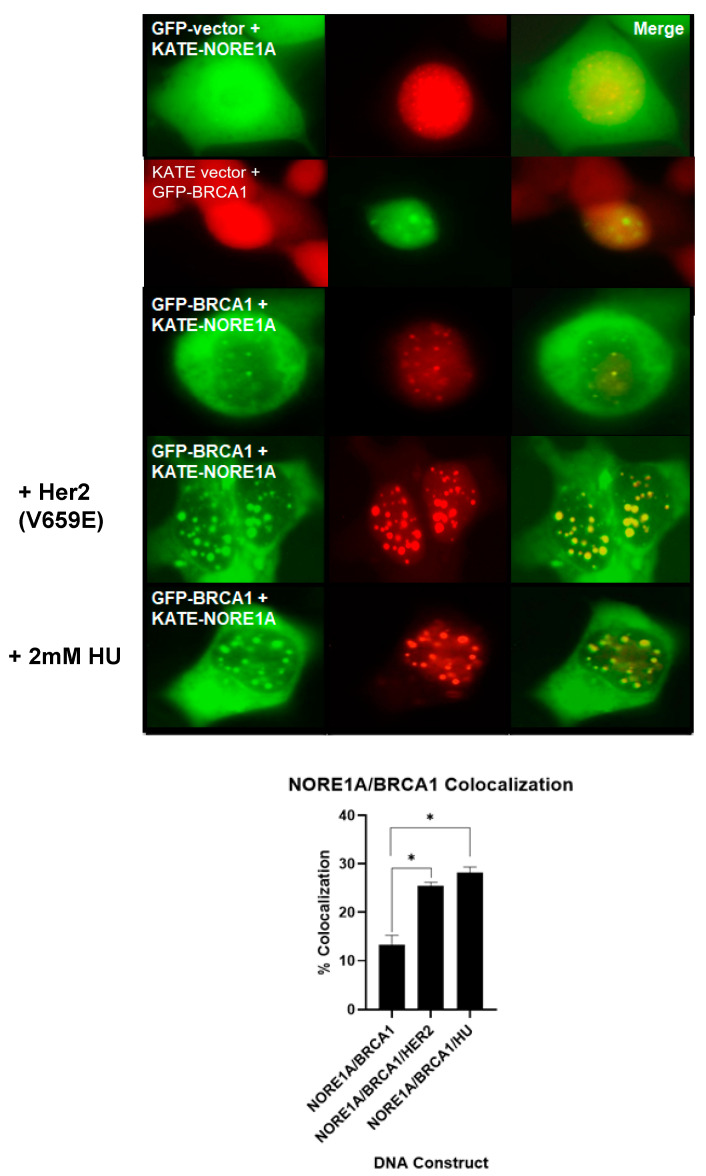
Co-localization of NORE1A and BRCA1 in nuclear speckles. HEK-293T cells were transfected with GFP-vector or GFP-tagged BRCA1 and RFP (KATE)-NORE1A, in the presence or absence of activated Her2 (V659E), or 2 mM hydroxyurea (HU) for 6 h. Images were captured using a fluorescence microscope (magnification = 1000×). Quantification is shown below. Statistically significant differences are marked with *.

Co-transfection experiments showed that we could detect weak, partial co-localization of the fluorescently tagged NORE1A and BRCA1 proteins in live HEK-293 cells (Figure 1). The co-localization in nuclear dots was dramatically enhanced in the presence of activated Her2, simulating Her2 overexpression, where the dots become both more numerous and larger (Figure 1). BRCA1 localizes to sites of replication fork stress [15,17], where it serves to stabilize the fork and assemble a complex to induce homologous repair of any double-strand breaks. To determine if the dots represented sites of replication fork arrest, we treated the cells with hydroxy urea (HU), which promotes replication fork stalling [52]. In treated cells, the co-localized nuclear dots were strikingly enhanced, suggesting the site of NORE1A/BRCA1 interaction is the stalled replication fork (Figure 1).

**Figure 2:**
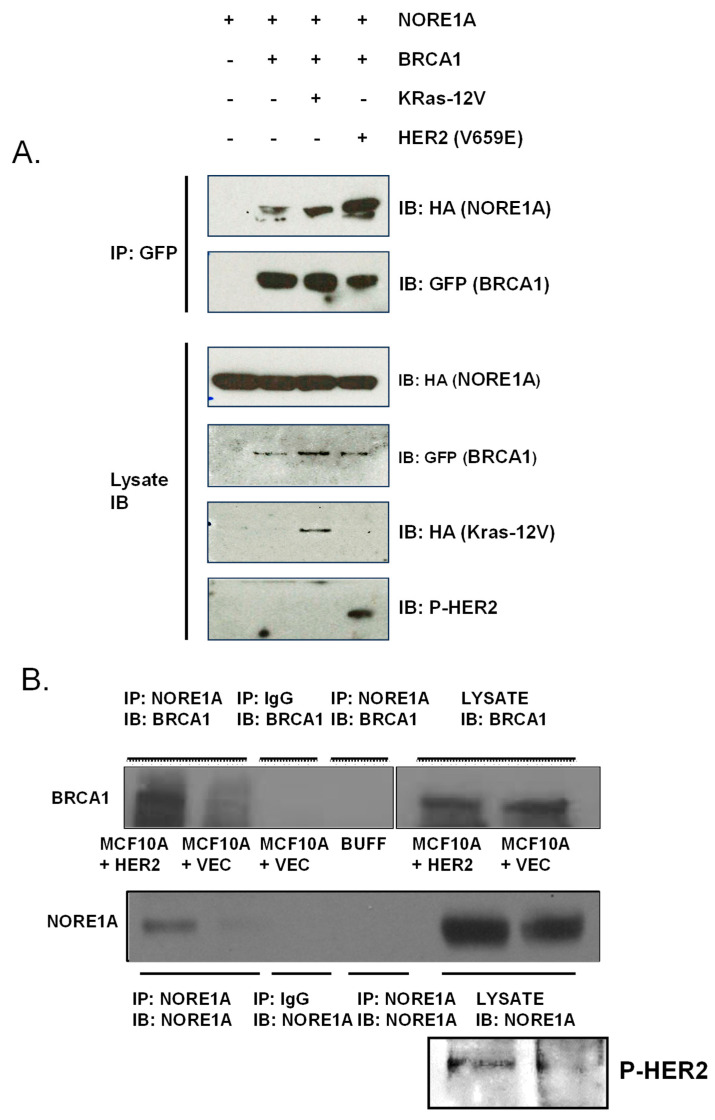

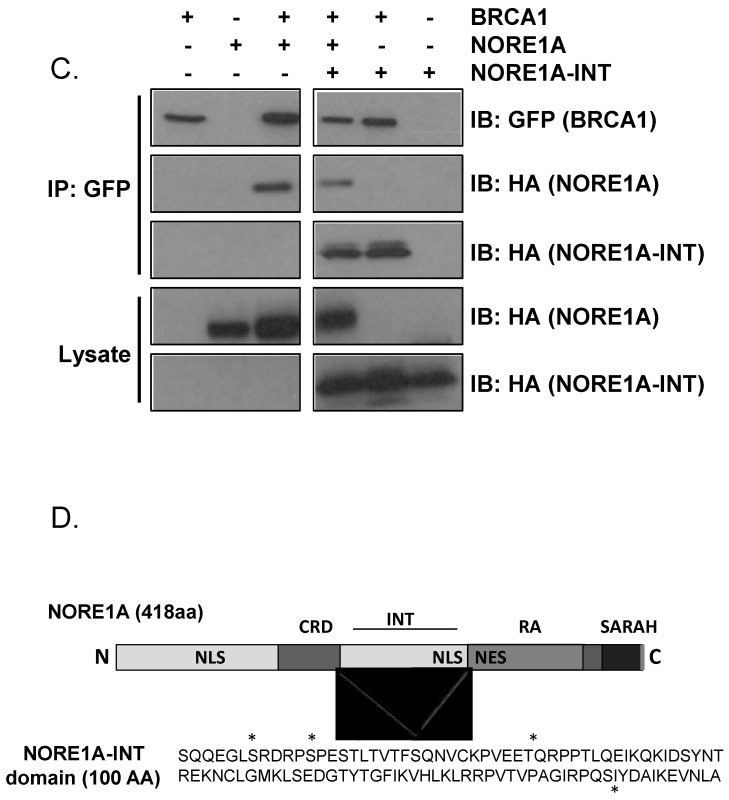
NORE1A and BRCA1 form a complex. (**A**) HEK-293T cells were transiently transfected with either GFP-tagged BRCA1 or HA-tagged NORE1A in the presence or absence of K-Ras12V or mutant Her2 (V659E) expression constructs. Cells were lysed 24 h after transfection and equal amounts of protein were immunoprecipitated for GFP. The immunoprecipitate was fractionated on an SDS-polyacrylamide gel and then immunoblotted with anti-HA and antibodies. (**B**) Equal amounts of protein lysate from MCF-10A human breast epithelial cells stably transfected with Her2 (V659E) or a vector control were immunoprecipitated (IP) with anti-NORE1A antibody, fractionated on an SDS-polyacrylamide gel, and then immunoblotted (IB) for the presence of BRCA1 in the complex. Cell lysate immunoprecipitated with IgG alone and lysis buffer immunoprecipitated with NORE1A antibody were used as negative controls. Her2 activity in the mutant Her2 transfected cells was confirmed using a phospho-Her2 antibody. (**C**) Full-length NORE1A or the INT fragment of NORE1A were co-transfected into HEK-293T cells with GFP-BRCA1 and co-immunoprecipitated with GFP. NORE1A/INT input levels are shown in the lysate control panels. The left set of panels shows the full-length NORE1A control IP. The right set shows the IP with INT. (**D**) Diagram showing the INT domain of NORE1A. * represents potential consensus sites of S/T phosphorylation. Raw blots from which the figures were derived are shown in Supplementary Materials File S1.

We confirmed that the co-localization observed in Figure 1 represented a complex formation by performing co-immunoprecipitation studies of the differentially tagged, overexpressed proteins. The proteins co-immunoprecipitated, and this effect was increased in the presence of either activated K-RAS or mutant Her2 (Figure 2A).

To confirm that the interaction was of physiological relevance, we performed co-immunoprecipitations of the endogenous proteins from vector or activated Her2 stably transfected MCF-10A breast cells. Figure 2B shows that the endogenous proteins form a complex and that this complex is enhanced by the presence of activated Her2.

To map the site of interaction of NORE1A and BRCA1, we performed deletion mutagenesis of NORE1A and determined that the domain lying between the CRD (cysteine-rich domain) and the RA (RAS association) domain was sufficient for the interaction (Figure 2C). We refer to this domain as the intermediate or INT domain. This domain is approximately 100 amino acids in length and contains multiple sites of potential phosphorylation that might be involved in regulating the interaction (Figure 2D).

### 3.2. Dual Inhibition of NORE1A and BRCA1 Has a Cooperative Effect on Transformation

**Figure 3:**
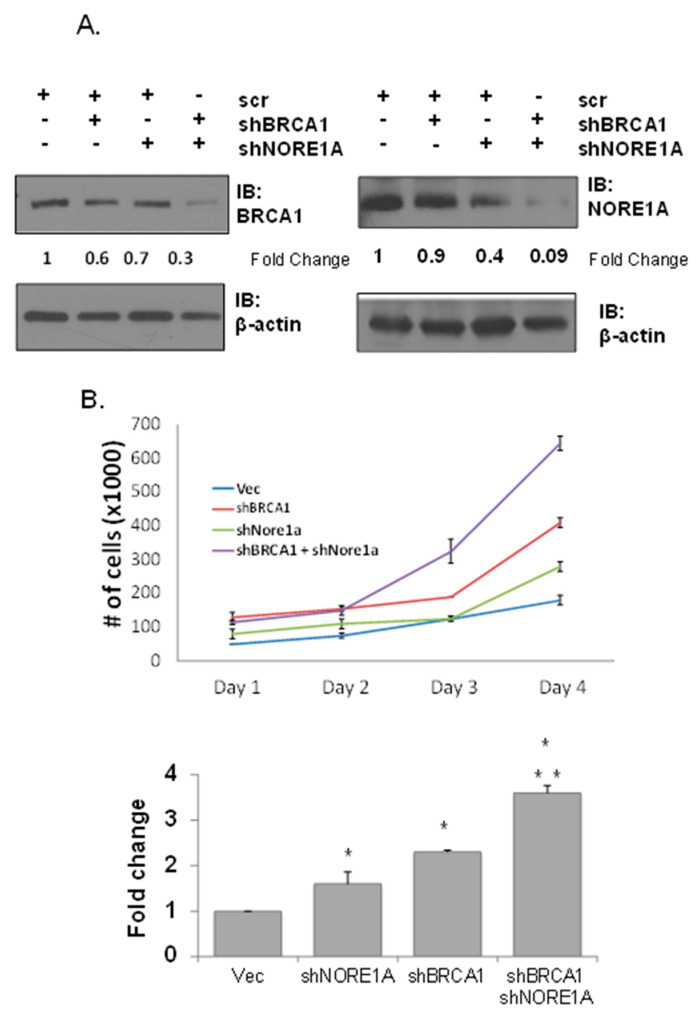
The effects of NORE1A/BRCA1 knockdown on MCF-10A cells. (**A**) Western blot analysis of NORE1A and BRCA1 expression in MCF-10A immortalized human mammary epithelial cells was performed. Expression levels of NORE1A and BRCA1 were quantified after sequential stable transduction of shRNA against NORE1A or BRCA1 or a scrambled control into these cells. The density of the bands was quantified using ImageJ software and relative BRCA1 or NORE1A expression was calculated after normalizing to β-actin expression to confirm knockdown (numbers shown under blots). (**B**) Growth curves of MCF-10A cells knocked down for NORE1A/BRCA1. Matched sets of knockdown MCF-10A cells were plated at 2 × 10^4^ and grown in 2D culture for 4 days. Cells were trypsinized and counted each day. (bottom) Relative changes in growth between each set on Day 4 expressed as a bar graph. Data represent the mean ± SD of duplicate experiments. Data that are significantly different are indicated by asterisks as follows: *, *p* < 0.05 compared to vector control; **, *p* < 0.05 compared to shNORE1A (Student’s *t*-test).

To examine the biological consequences of the NORE1A loss of expression in BRCA1 defective breast cells, we used shRNAs against the two genes to create a matched set of stable cell lines derived from the non-transformed MCF-10A breast cell line [53] (Figure 3). The suppression of protein expression was confirmed by Western analysis (Figure 3, top). The matched set of cell lines was then compared for any effects on growth. While suppression of either tumor suppressor alone had a modest effect on enhancing the growth rate, this was greater than additive in the double knockdown cells (Figure 3).

**Figure 4:**
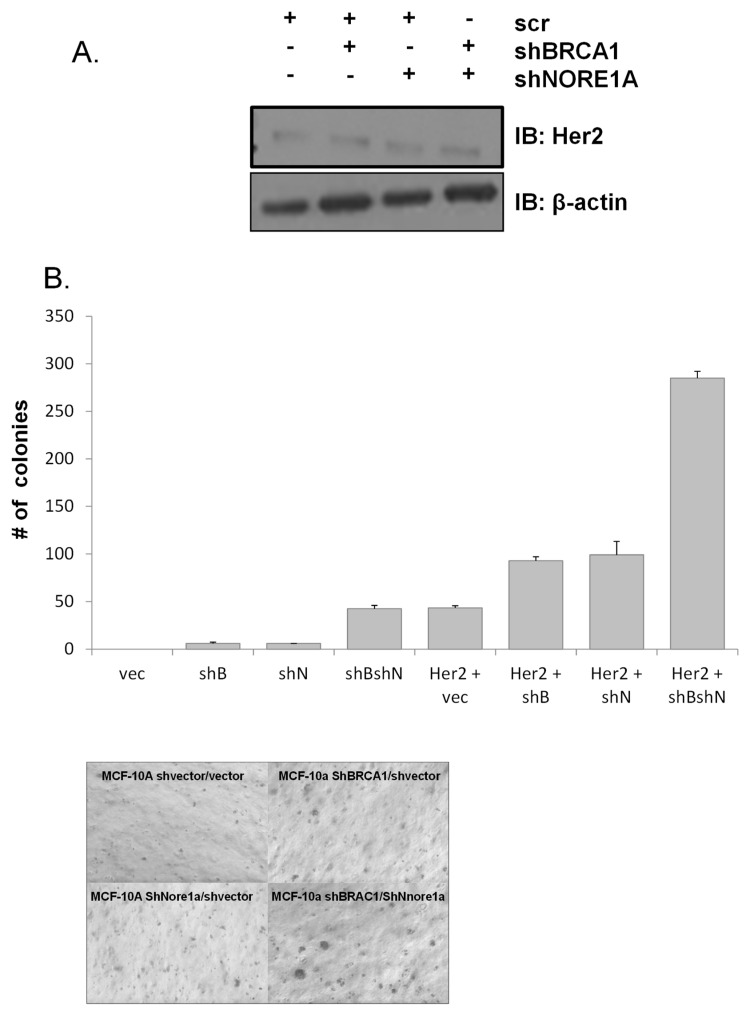
Effects of NORE1A/BRCA1 knockdown on Her2 mediated transformation. (**A**) Western blot analysis of Her2 expression in shBRCA1/shNORE1A MCF-10A cells. Her2 (V659E) was stably overexpressed in the matched set of MCF-10A knockdown cells from (**A**) and its expression was validated by Western blot. (**B**) Soft agar assays: shBRCA1 (shB) and shNORE1A (shN) MCF-10A cells with and without Her2-overexpression (Her2+) were plated in soft agar and grown for 2 weeks at 37 °C. Data represent the mean ± SD of 3 independent experiments. A representative picture of a soft agar assay is shown below.

To determine the effects in a Her2-positive cell system, the cells were then further transfected with activated Her2 to generate a stable Her2+, BRCA1+/−, NORE1A+/− matched set cell system (Figure 4). We then assayed the cells for in vitro transformation using soft agar assays. The suppression of either tumor suppressor resulted in the growth of a small number of colonies, which was cooperatively increased when both were inactivated together. Her2-transfected shRNA vector cells formed colonies, and this effect was enhanced by a single tumor suppressor knockdown. It was enhanced further in the dual tumor suppressor knockdown system (Figure 4).

### 3.3. NORE1A Is Essential for BRCA1 Loss-Induced Senescence

**Figure 5:**
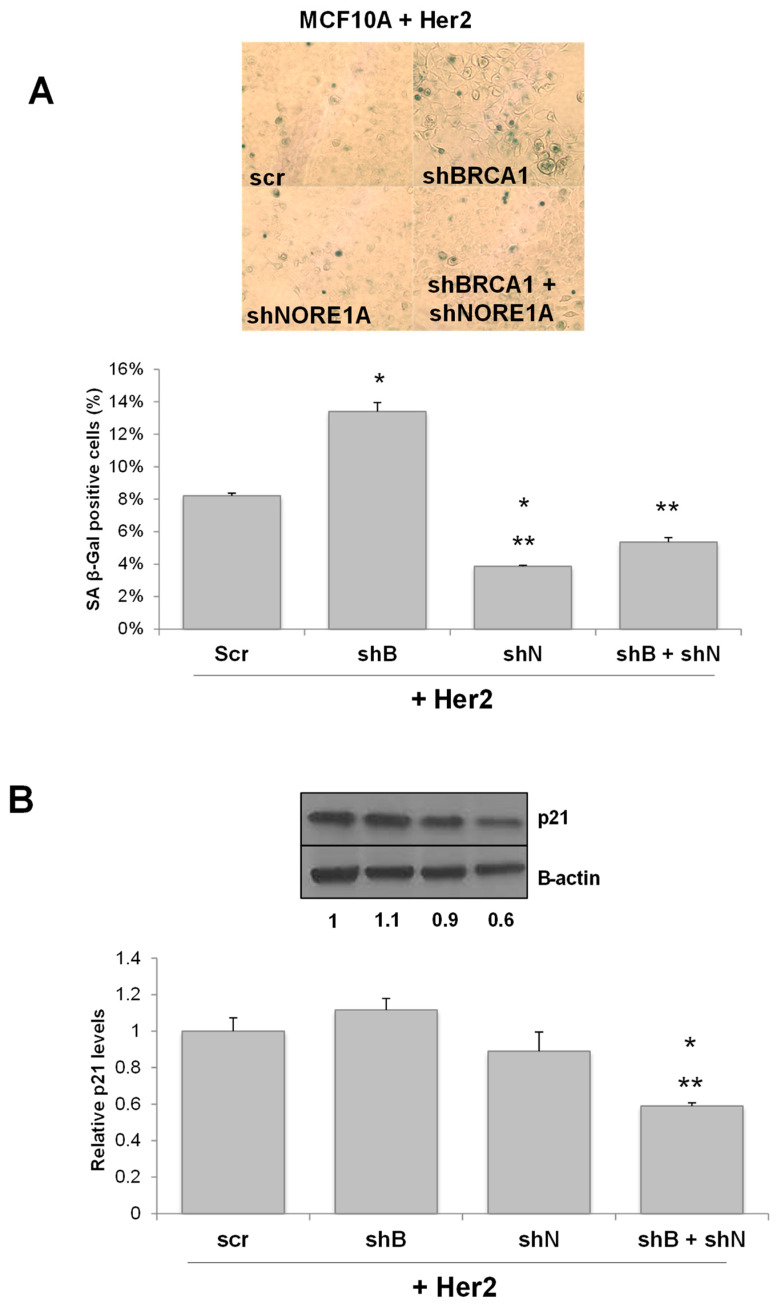
Effects of NORE1A/BRCA1 expression on senescence of Her2-transformed cells. (**A**) shBRCA1 (shB) and shNORE1A (shN) MCF-10A Her2+ cells were grown in 12-well plates and β-galactosidase activity was quantified after 24 h. The percent increase over Her2+ control cells was measured. Data represent the mean ± SD of triplicate experiments. *, *p* < 0.05 compared to scrambled control; **, *p* < 0.05 compared to shBRCA1 (Student’s *t*-test). (**Right**) A representative image of senescence induction in each set of cells. (**B**) Double knockdown of NORE1A/BRCA1 cooperatively suppresses p21CIP. (**Right**) Western blot analysis of p21CIP expression from shBRCA1/shNORE1A MCF-10A Her2+ cells. The density of the bands was quantified using ImageJ software and relative p21CIP expression was quantified after normalizing to β-actin. (**Left**) Relative p21 levels expressed as a bar graph. Data represent the mean ± SD of three independent experiments. *, *p* < 0.05 compared to scrambled control; **, *p* < 0.05 compared to shBRCA1 (students *t*-test).

Although inactivation of BRCA1 is a key driver of many breast cancers, counter-intuitively, the initial biological effects of BRCA1 inactivation can be haploinsufficient induction of senescence or HIS [22]. The activation of Her2 can also promote senescence [35], likely via its activation of RAS. Additional genetic lesions may be required to overcome these senescence defense mechanisms to facilitate full malignancy. In the matched set Her2+ cell system, the inactivation of BRCA1 indeed resulted in an increase in B-galactosidase staining above the background due to the presence of Her2 alone (Figure 5A). However, the inactivation of NORE1A suppressed the basal levels of senescence. When both NORE1A and BRCA1 were suppressed, the NORE1A effect was dominant, reducing the senescence of the Her2+/BRCA1− cells by over half. Both BRCA1 [54] and NORE1A [55] can upregulate the expression of the cell cdk inhibitor p21CIP to promote cell cycle arrest, and this can serve as an additional indicator of senescence. The knockdown of both BRCA1 and NORE1A caused the largest decrease in p21CIP1 levels in the matched cell set (Figure 5B).

### 3.4. BRCA1 Is Essential for NORE1A-Induced Senescence

**Figure 6:**
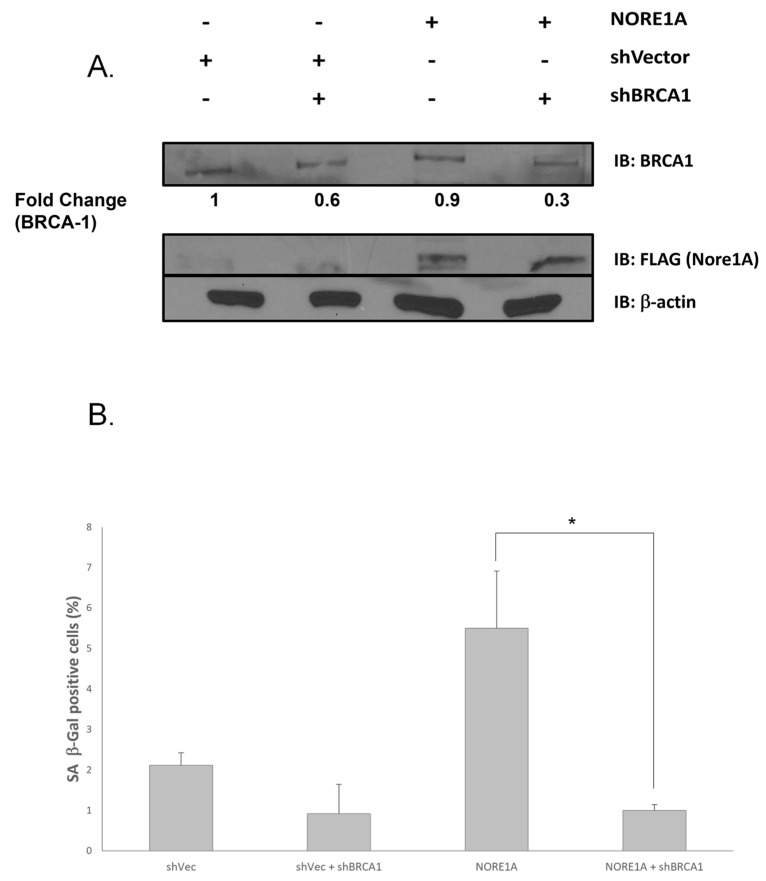
Overexpression of NORE1A induces BRCA1 dependent senescence in MCF-7 cells. (**A**) MCF-7 cells were stably transfected with shRNA to BRCA1 and/or an HA-tagged NORE1A expression plasmid. Protein expression was validated by Western blot in the matched set of cell lines. BRCA1 knockdown was confirmed by quantification relative to actin. (**B**) The cells were assayed for senescence by B-Galactosidase staining. * Indicates *p* value < 0.05.

To confirm the link between NORE1A and BRCA1 in senescence, we performed the reciprocal experiment. We generated a matched cell set of MCF-7 cells, which are normally NORE1A negative [45] and BRCA1 positive [56]. We knocked down BRCA1 and overexpressed NORE1A from a CMV-based promoter. After validating the expression levels of the proteins in each cell line (Figure 6A), we then examined the levels of senescence in the system (Figure 6B). The overexpression of NORE1A induced considerable senescence (as measured by B Galactosidase activity), which was almost completely abrogated in the BRCA1 shRNA knockdown cells.

## 4. Discussion

Breast cancer is often driven by the upregulation of the Her2 oncoprotein or inactivation of the BRCA1 tumor suppressor [2,3,4,5,30,31]. In some cases, breast tumors can exhibit both genetic defects and these Her2+/BRCA1 negative tumors tend to exhibit worse prognosis [38,39,40].

The activation of oncogenes, such as Her2, promotes genomic instability by enhancing the rate of cellular proliferation to the point where normal nucleotide pools become depleted. This impedes the progression of DNA replication forks [57]. Persistently stalled forks can collapse, leading to the formation of DSBs [58]. The subsequent replication stress and DNA damage accumulation drive the activation of a senescence response [59].

Inactivation of the BRCA1 tumor suppressor, which normally acts at stalled replication forks to mediate the formation of complexes that promote stabilization and DNA repair [15,16,17,18], leads to enhanced DNA damage. This can also result in the activation of a DDR senescence response. This effect has been described as haploinsufficient senescence, or (HIS) [22].

It has been postulated that this oncogene-induced (OIS) and haploinsufficiency-induced senescence (HIS) are evolved responses to repress cancer development [60,61]. As tumors progress, the senescence tends to decrease [62], and experimentally cells can escape from senescence induction by the inactivation of components of the p53 and Rb tumor suppressor pathways [63]. Thus, progression toward malignancy after Her2 activation or BRCA1 inactivation may require the acquisition of additional genetic defects that abrogate senescence induction. This may be particularly important for breast tumors that are both Her2+ and deficient for BRCA1.

NORE1A is a RAS effector that is also a tumor suppressor [55]. It can modulate the P53/p21 pathway by inducing specific post-translational modifications of p53 to promote senescence [43]. It can also modulate Rb phosphorylation to promote Rb-dependent senescence [44]. Therefore, it is linked to the machinery known to be involved in both OIS and HIS. NORE1A is often downregulated in breast cancer [45] by a process of promoter methylation. Indeed, our database analysis shows that there is a statistically significant relationship (*p* < 0.05, r = −0.27) between the reduced expression of BRCA1 and the reduced expression of NORE1A in Her2+, but not Her− breast cancers (Breast Cancer Gene-Expression Miner v3.2) [46]. Therefore, we hypothesized that a loss of NORE1A could explain how Her2+/BRCA1− breast tumors overcome the OIS/HIS senescence responses.

Our initial experiments suggested a much closer relationship between NORE1A and BRCA1 than we expected, as we observed that NORE1A and BRCA1 could co-localize in the same nuclear speckle structures. These speckles may be sites of replication fork stalling as treatment with HU (a fork stalling inducer) dramatically enhanced the effect. We then performed co-immunoprecipitation studies that confirmed the two proteins could form a stable complex and that the complex could be regulated by an activated Her2 mutant or activated RAS. We were able to confirm the complex formation is physiological by repeating the experiments on the endogenous proteins. Mapping studies showed that the region between the CRD region and the RAS-association domain of NORE1A (which we have named INT for the intermediate region) was essential for the interaction. This region could be exposed when RAS binds to the Ras-association domain of NORE1A [64], providing a mechanistic explanation for the observation that the interaction is increased by RAS activation.

When we examined the effects of re-expressing NORE1A in NORE1A-negative MCF-7 breast tumor cells, we found that its ability to induce senescence was impaired if we knocked down BRCA1. Thus, we identified a novel NORE1A/BRCA1 tumor suppressor complex and identified a new link between Her2 and BRCA1 via NORE1A.

Deciphering the function of scaffolding proteins, such as NORE1A, using overexpression studies is fraught with difficulties in the interpretation of the results, as inducing incorrect stoichiometry could have an inhibitory rather than a stimulatory result. Therefore, to confirm the role of NORE1A suppression on BRCA1 activity, we generated a set of matched cells derived from the non-transformed MCF-10A breast cell line. The inactivation of either tumor suppressor enhanced the cellular growth rate, but this was even further enhanced by the dual inactivation of both tumor suppressors. Moreover, a cooperative increase in the ability to form colonies in soft agar was observed when both proteins were suppressed. The addition of Her2 activation to the system further enhanced the effect. When we quantified senescence levels in the Her2 transformed cells, knocking down BRCA1 alone caused an increase (HIS), but this was abrogated when NORE1A was also suppressed. Thus, NORE1A forms a tumor suppressor complex with BRCA1, and NORE1A loss uncouples Her2+/BRCA1− cells from senescence induction. We noticed that knocking down NORE1A also seemed to reduce the levels of BRCA1 protein. It is possible that NORE1A may have a stabilizing effect on BRCA1.

Like BRCA1, BRCA2 plays a key role in stabilizing replication forks and maintaining genomic stability, although via distinct a mechanism [65]. The inactivation of BRCA2 can also promote senescence [66]. The RASSF1A tumor suppressor is closely related to NORE1A and has been shown to regulate BRCA2 activity via the HIPPO pathway [67]. As NORE1A can also activate the HIPPO pathway [68], it is possible that NORE1A could modulate both BRCA1 and BRCA2.

Classically, senescence involves permanent growth arrest. However, it is to be noted that the role of senescence in cancer is complex, as some tumor cells can develop many of the characteristics of senescence while retaining proliferative capacity. This state has been described as SWING (senescence with incomplete growth arrest) [69]. These SWING cells exhibit a senescence-associated secretory phenotype (SASP) and produce inflammatory cytokines, which can modulate the tumor microenvironment to support rather than suppress malignancy and metastasis [35,48]. The role of NORE1A in these processes remains a question for future investigation.

## 5. Conclusions

Here we identify a novel signaling pathway between RAS and BRCA1 via NORE1A, which may have an important role in the OIS and HIS classes of senescence. We show why the loss of NORE1A expression in breast cancers may be particularly important for the development of tumors that are BRCA1 deficient and Her2 positive. It also suggests that the suppression of NORE1A may have effects on replication fork stability and hence the development of DNA damage.

## Data Availability

Not applicable.

## Supplementary Materials

The following supporting information can be downloaded at: https://www.mdpi.com/article/10.3390/cancers15164133/s1.

## References

[1] King M.C., Marks J.H., Mandell J.B. (2003). Breast and ovarian cancer risks due to inherited mutations in BRCA1 and BRCA2. Science.

[2] Magdinier F., Ribieras S., Lenoir G.M., Frappart L., Dante R. (1998). Down-regulation of BRCA1 in human sporadic breast cancer; analysis of DNA methylation patterns of the putative promoter region. Oncogene.

[3] Rice J.C., Futscher B.W. (2000). Transcriptional repression of BRCA1 by aberrant cytosine methylation, histone hypoacetylation and chromatin condensation of the BRCA1 promoter. Nucleic Acids Res..

[4] Futreal P.A., Liu Q., Shattuck-Eidens D., Cochran C., Harshman K., Tavtigian S., Bennett L.M., Haugen-Strano A., Swensen J., Miki Y. (1994). BRCA1 mutations in primary breast and ovarian carcinomas. Science.

[5] Katsama A., Sourvinos G., Zachos G., Spandidos D.A. (2000). Allelic loss at the BRCA1, BRCA2 and TP53 loci in human sporadic breast carcinoma. Cancer Lett..

[6] Takaoka M., Miki Y. (2018). BRCA1 gene: Function and deficiency. Int. J. Clin. Oncol..

[7] Quinn J.E., Kennedy R.D., Mullan P.B., Gilmore P.M., Carty M., Johnston P.G., Harkin D.P. (2003). BRCA1 functions as a differential modulator of chemotherapy-induced apoptosis. Cancer Res..

[8] Chen Q., Lei J.H., Bao J., Wang H., Hao W., Li L., Peng C., Masuda T., Miao K., Xu J. (2020). BRCA1 Deficiency Impairs Mitophagy and Promotes Inflammasome Activation and Mammary Tumor Metastasis. Adv. Sci..

[9] Sankaran S., Starita L.M., Groen A.C., Ko M.J., Parvin J.D. (2005). Centrosomal microtubule nucleation activity is inhibited by BRCA1-dependent ubiquitination. Mol. Cell Biol..

[10] Rosen E.M., Fan S., Ma Y. (2006). BRCA1 regulation of transcription. Cancer Lett..

[11] Bochar D.A., Wang L., Beniya H., Kinev A., Xue Y., Lane W.S., Wang W., Kashanchi F., Shiekhattar R. (2000). BRCA1 is associated with a human SWI/SNF-related complex: Linking chromatin remodeling to breast cancer. Cell.

[12] Tu Z., Aird K.M., Zhang R. (2013). Chromatin remodeling, BRCA1, SAHF and cellular senescence. Cell Cycle.

[13] Savage K.I., Harkin D.P. (2015). BRCA1, a ‘complex’ protein involved in the maintenance of genomic stability. FEBS J..

[14] Caestecker K.W., Van de Walle G.R. (2013). The role of BRCA1 in DNA double-strand repair: Past and present. Exp. Cell Res..

[15] Powell S.N., Kachnic L.A. (2003). Roles of BRCA1 and BRCA2 in homologous recombination, DNA replication fidelity and the cellular response to ionizing radiation. Oncogene.

[16] Zhang J., Powell S.N. (2005). The role of the BRCA1 tumor suppressor in DNA double-strand break repair. Mol. Cancer Res..

[17] Long D.T., Joukov V., Budzowska M., Walter J.C. (2014). BRCA1 promotes unloading of the CMG helicase from a stalled DNA replication fork. Mol. Cell.

[18] Daza-Martin M., Starowicz K., Jamshad M., Tye S., Ronson G.E., MacKay H.L., Chauhan A.S., Walker A.K., Stone H.R., Beesley J.F.J. (2019). Isomerization of BRCA1-BARD1 promotes replication fork protection. Nature.

[19] Ongusaha P.P., Ouchi T., Kim K.T., Nytko E., Kwak J.C., Duda R.B., Deng C.X., Lee S.W. (2003). BRCA1 shifts p53-mediated cellular outcomes towards irreversible growth arrest. Oncogene.

[20] Deng C.X. (2006). BRCA1: Cell cycle checkpoint, genetic instability, DNA damage response and cancer evolution. Nucleic Acids Res..

[21] Cao L., Li W., Kim S., Brodie S.G., Deng C.X. (2003). Senescence, aging, and malignant transformation mediated by p53 in mice lacking the Brca1 full-length isoform. Genes Dev..

[22] Sedic M., Skibinski A., Brown N., Gallardo M., Mulligan P., Martinez P., Keller P.J., Glover E., Richardson A.L., Cowan J. (2015). Haploinsufficiency for BRCA1 leads to cell-type-specific genomic instability and premature senescence. Nat. Commun..

[23] Zhu H., Blake S., Kusuma F.K., Pearson R.B., Kang J., Chan K.T. (2020). Oncogene-induced senescence: From biology to therapy. Mech. Ageing Dev..

[24] Serrano M., Lin A.W., McCurrach M.E., Beach D., Lowe S.W. (1997). Oncogenic ras provokes premature cell senescence associated with accumulation of p53 and p16INK4a. Cell.

[25] Yaswen P., Campisi J. (2007). Oncogene-induced senescence pathways weave an intricate tapestry. Cell.

[26] Gimple R.C., Wang X. (2019). RAS: Striking at the Core of the Oncogenic Circuitry. Front. Oncol..

[27] Galie M. (2019). RAS as Supporting Actor in Breast Cancer. Front. Oncol..

[28] Clark G.J., Der C.J. (1995). Aberrant function of the Ras signal transduction pathway in human breast cancer. Breast Cancer Res. Treat..

[29] Eckert L.B., Repasky G.A., Ulku A.S., McFall A., Zhou H., Sartor C.I., Der C.J. (2004). Involvement of Ras activation in human breast cancer cell signaling, invasion, and anoikis. Cancer Res..

[30] Bose R., Kavuri S.M., Searleman A.C., Shen W., Shen D., Koboldt D.C., Monsey J., Goel N., Aronson A.B., Li S. (2013). Activating HER2 mutations in HER2 gene amplification negative breast cancer. Cancer Discov..

[31] Pegram M.D., Konecny G., Slamon D.J. (2000). The molecular and cellular biology of HER2/neu gene amplification/overexpression and the clinical development of herceptin (trastuzumab) therapy for breast cancer. Cancer Treat. Res..

[32] Goodearl A., Viehover A., Vartanian T. (2001). Neuregulin-induced association of Sos Ras exchange protein with HER2(erbB2)/HER3(erbB3) receptor complexes in Schwann cells through a specific Grb2-HER2(erbB2) interaction. Dev. Neurosci..

[33] Von Lintig F.C., Dreilinger A.D., Varki N.M., Wallace A.M., Casteel D.E., Boss G.R. (2000). Ras activation in human breast cancer. Breast Cancer Res. Treat..

[34] Trost T.M., Lausch E.U., Fees S.A., Schmitt S., Enklaar T., Reutzel D., Brixel L.R., Schmidtke P., Maringer M., Schiffer I.B. (2005). Premature senescence is a primary fail-safe mechanism of ERBB2-driven tumorigenesis in breast carcinoma cells. Cancer Res..

[35] Angelini P.D., Zacarias Fluck M.F., Pedersen K., Parra-Palau J.L., Guiu M., Bernado Morales C., Vicario R., Luque-Garcia A., Navalpotro N.P., Giralt J. (2013). Constitutive HER2 signaling promotes breast cancer metastasis through cellular senescence. Cancer Res..

[36] Sinha B., Song K. (1997). Role of ras oncogene in adriamycin resistance in human prostate tumor cells. Int. J. Oncol..

[37] Wagner V., Gil J. (2020). Senescence as a therapeutically relevant response to CDK4/6 inhibitors. Oncogene.

[38] Omarini C., Bettelli S., Caprera C., Manfredini S., Caggia F., Guaitoli G., Moscetti L., Toss A., Cortesi L., Kaleci S. (2018). Clinical and molecular predictors of long-term response in HER2 positive metastatic breast cancer patients. Cancer Biol. Ther..

[39] Reed W., Sandstad B., Holm R., Nesland J.M. (2003). The prognostic impact of hormone receptors and c-erbB-2 in pregnancy-associated breast cancer and their correlation with BRCA1 and cell cycle modulators. Int. J. Surg. Pathol..

[40] Ansquer Y., Mandelbrot L., Lehy T., Salomon L., Dhainaut C., Madelenat P., Feldmann G., Walker F. (2005). Expression of BRCA1, HER-1 (EGFR) and HER2 in sporadic breast cancer and relationships to other clinicopathological prognostic features. Anticancer Res..

[41] Vos M.D., Martinez A., Ellis C.A., Vallecorsa T., Clark G.J. (2003). The pro-apoptotic Ras effector Nore1 may serve as a Ras-regulated tumor suppressor in the lung. J. Biol. Chem..

[42] Donninger H., Barnoud T., Clark G.J. (2016). NORE1A is a double barreled Ras senescence effector that activates p53 and Rb. Cell Cycle.

[43] Donninger H., Calvisi D.F., Barnoud T., Clark J., Schmidt M.L., Vos M., Clark G. (2014). NORE1A is a Ras senescence effector that controls the apoptotic/senescent balance of p53 via HIPK2. J. Cell Biol..

[44] Barnoud T., Donninger H., Clark G.J. (2015). Ras regulates Rb via NORE1A. J. Biol. Chem..

[45] Hesson L., Dallol A., Minna J.D., Maher E.R., Latif F. (2003). NORE1A, a homologue of RASSF1A tumour suppressor gene is inactivated in human cancers. Oncogene.

[46] Jezequel P., Frenel J.S., Campion L., Guerin-Charbonnel C., Gouraud W., Ricolleau G., Campone M. (2013). bc-GenExMiner 3.0: New mining module computes breast cancer gene expression correlation analyses. Database.

[47] Li Y.M., Pan Y., Wei Y., Cheng X., Zhou B.P., Tan M., Zhou X., Xia W., Hortobagyi G.N., Yu D. (2004). Upregulation of CXCR4 is essential for HER2-mediated tumor metastasis. Cancer Cell.

[48] Zacarias-Fluck M.F., Morancho B., Vicario R., Luque Garcia A., Escorihuela M., Villanueva J., Rubio I.T., Arribas J. (2015). Effect of cellular senescence on the growth of HER2-positive breast cancers. J. Natl. Cancer Inst..

[49] Tu Z., Aird K.M., Zhang R. (2012). RAS, cellular senescence and transformation: The BRCA1 DNA repair pathway at the crossroads. Small GTPases.

[50] Barnoud T., Schmidt M.L., Donninger H., Clark G.J. (2017). The role of the NORE1A tumor suppressor in Oncogene-Induced Senescence. Cancer Lett..

[51] Scully R., Chen J., Ochs R.L., Keegan K., Hoekstra M., Feunteun J., Livingston D.M. (1997). Dynamic changes of BRCA1 subnuclear location and phosphorylation state are initiated by DNA damage. Cell.

[52] Petermann E., Orta M.L., Issaeva N., Schultz N., Helleday T. (2010). Hydroxyurea-stalled replication forks become progressively inactivated and require two different RAD51-mediated pathways for restart and repair. Mol. Cell.

[53] Qu Y., Han B., Yu Y., Yao W., Bose S., Karlan B.Y., Giuliano A.E., Cui X. (2015). Evaluation of MCF10A as a Reliable Model for Normal Human Mammary Epithelial Cells. PLoS ONE.

[54] Lee Y.H., Bedford M.T., Stallcup M.R. (2011). Regulated recruitment of tumor suppressor BRCA1 to the p21 gene by coactivator methylation. Genes Dev..

[55] Calvisi D.F., Donninger H., Vos M.D., Birrer M.J., Gordon L., Leaner V., Clark G.J. (2009). NORE1A tumor suppressor candidate modulates p21CIP1 via p53. Cancer Res..

[56] Buckley N.E., Nic An tSaoir C.B., Blayney J.K., Oram L.C., Crawford N.T., D’Costa Z.C., Quinn J.E., Kennedy R.D., Harkin D.P., Mullan P.B. (2013). BRCA1 is a key regulator of breast differentiation through activation of Notch signalling with implications for anti-endocrine treatment of breast cancers. Nucleic Acids Res..

[57] Duijf P.H.G., Nanayakkara D., Nones K., Srihari S., Kalimutho M., Khanna K.K. (2019). Mechanisms of Genomic Instability in Breast Cancer. Trends Mol. Med..

[58] Gaillard H., Garcia-Muse T., Aguilera A. (2015). Replication stress and cancer. Nat. Rev. Cancer.

[59] Di Micco R., Sulli G., Dobreva M., Liontos M., Botrugno O.A., Gargiulo G., dal Zuffo R., Matti V., d’Ario G., Montani E. (2011). Interplay between oncogene-induced DNA damage response and heterochromatin in senescence and cancer. Nat. Cell Biol..

[60] Munoz-Espin D., Serrano M. (2014). Cellular senescence: From physiology to pathology. Nat. Rev. Mol. Cell Biol..

[61] Narita M., Lowe S.W. (2005). Senescence comes of age. Nat. Med..

[62] Wyld L., Bellantuono I., Tchkonia T., Morgan J., Turner O., Foss F., George J., Danson S., Kirkland J.L. (2020). Senescence and Cancer: A Review of Clinical Implications of Senescence and Senotherapies. Cancers.

[63] Shay J.W., Pereira-Smith O.M., Wright W.E. (1991). A role for both RB and p53 in the regulation of human cellular senescence. Exp. Cell Res..

[64] Nussinov R., Zhang M., Tsai C.J., Liao T.J., Fushman D., Jang H. (2018). Autoinhibition in Ras effectors Raf, PI3Kalpha, and RASSF5: A comprehensive review underscoring the challenges in pharmacological intervention. Biophys. Rev..

[65] Roy R., Chun J., Powell S.N. (2012). BRCA1 and BRCA2: Different roles in a common pathway of genome protection. Nat. Rev. Cancer.

[66] Carlos A.R., Escandell J.M., Kotsantis P., Suwaki N., Bouwman P., Badie S., Folio C., Benitez J., Gomez-Lopez G., Pisano D.G. (2013). ARF triggers senescence in Brca2-deficient cells by altering the spectrum of p53 transcriptional targets. Nat. Commun..

[67] Pefani D.E., Latusek R., Pires I., Grawenda A.M., Yee K.S., Hamilton G., van der Weyden L., Esashi F., Hammond E.M., O’Neill E. (2015). RASSF1A-LATS1 signalling stabilizes replication forks by restricting CDK2-mediated phosphorylation of BRCA2. Nat. Cell Biol..

[68] Avruch J., Praskova M., Ortiz-Vega S., Liu M., Zhang X.F. (2005). Nore1 and RASSF1 Regulation of Cell Proliferation and of the MST1/2 Kinases. Methods Enzym..

[69] Sherman M.Y., Meng L., Stampfer M., Gabai V.L., Yaglom J.A. (2011). Oncogenes induce senescence with incomplete growth arrest and suppress the DNA damage response in immortalized cells. Aging Cell.

