# Supplementary material for: BRCA1 and NORE1A Form a Her2/Ras Regulated Tumor Suppressor Complex Modulating Senescence
Source: Cancers (Basel). 2023 Aug 16;15(16):4133. doi: 10.3390/cancers15164133 (PMC10452424; doi:10.3390/cancers15164133)

Supplementary Materials File S1

Figure 2 A

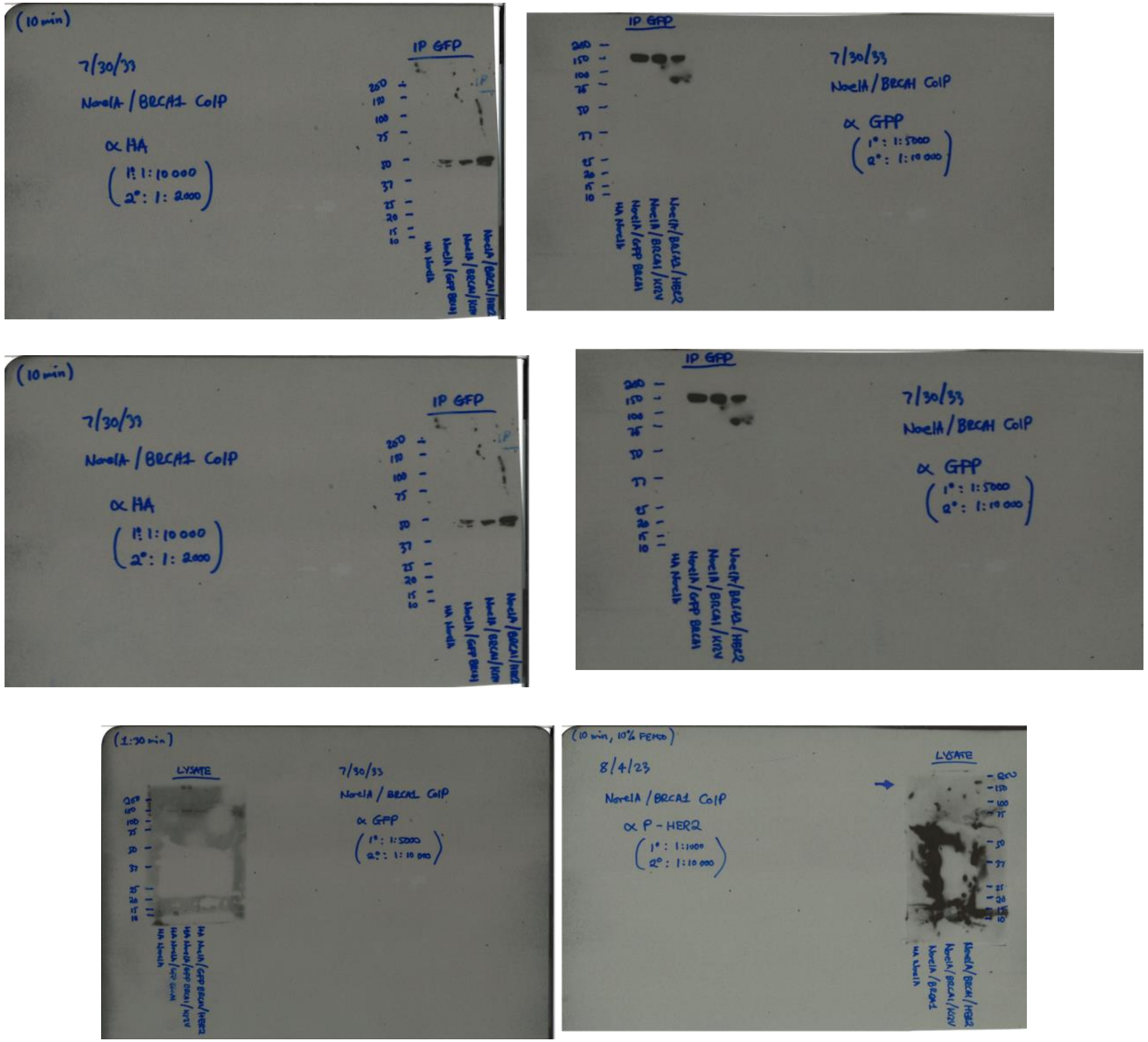

Figure 2B

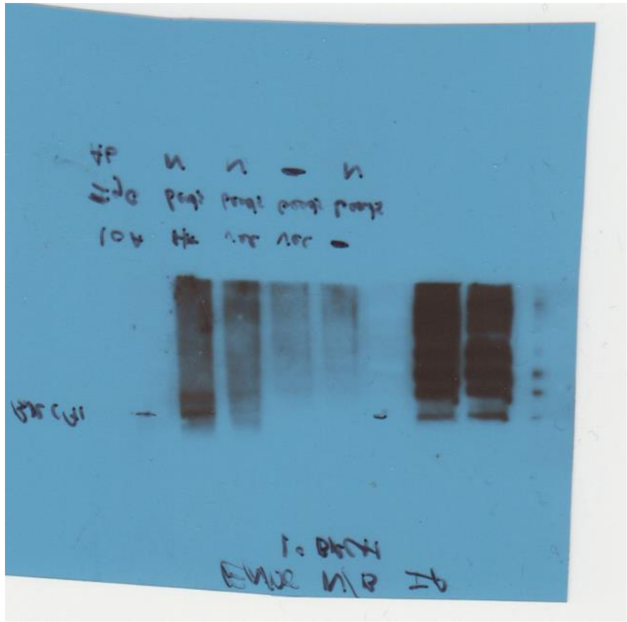

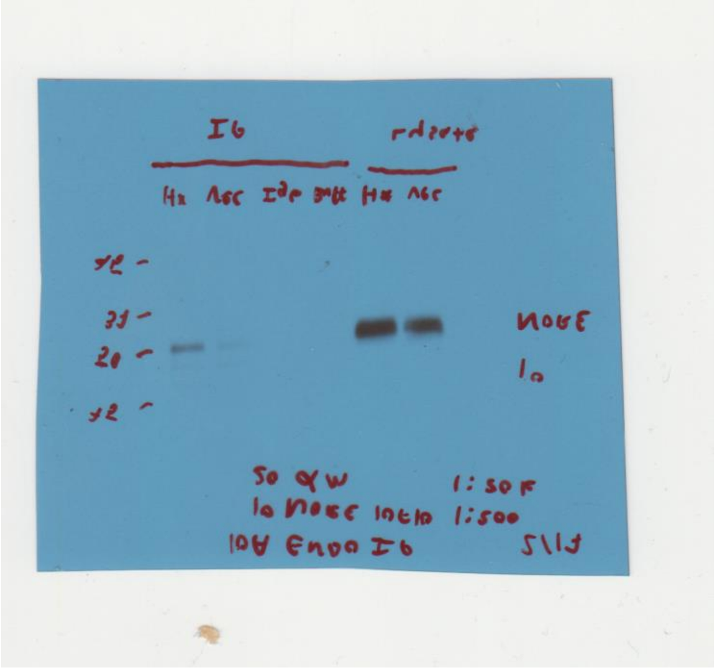

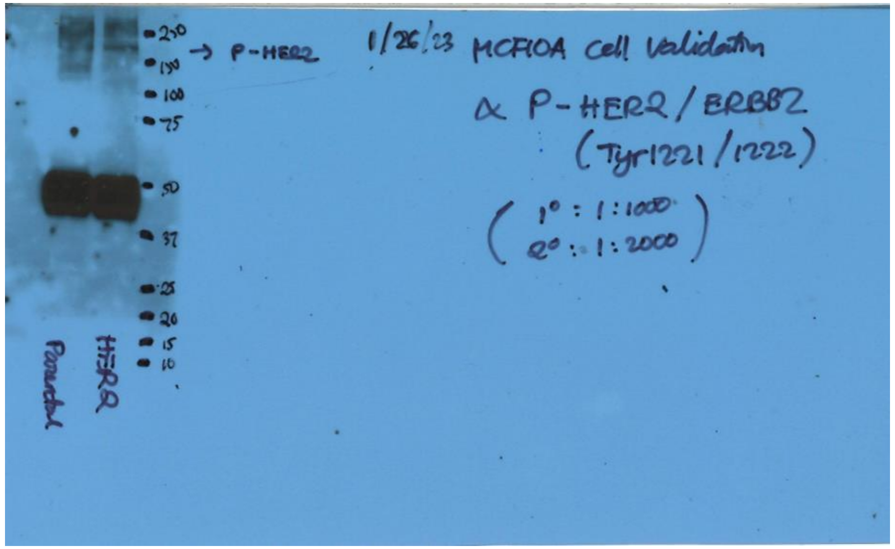
250
150
100
75
50
37
25
20
15
10
→ P-HER2
1/26/23 MCF10A Cell Validation
α P-HER2 / ERBB2
(Tyr1221/1222)
1° : 1:1000
2° : 1:2000
Parental
HER2

Figure 2C

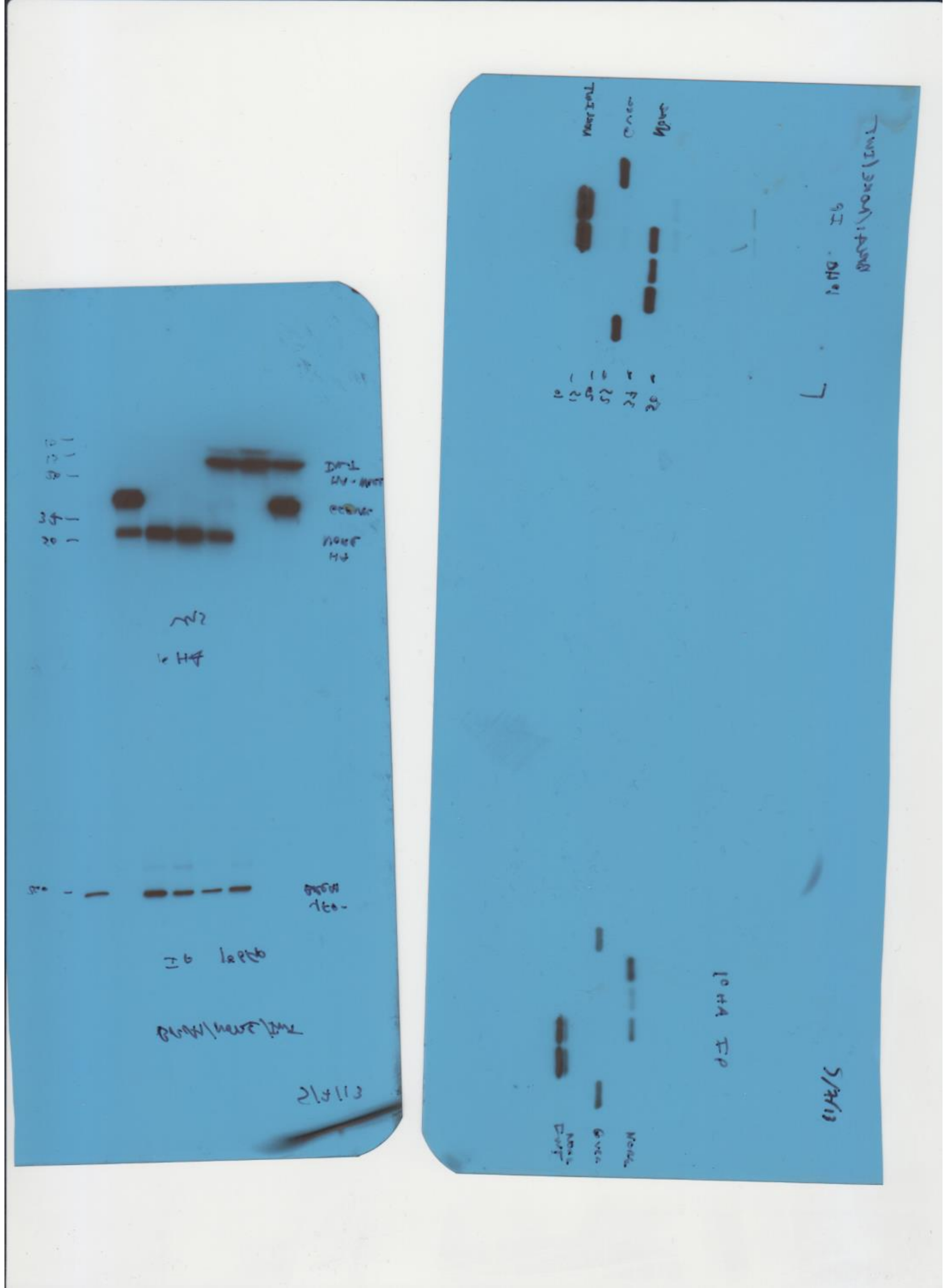

Figure 3

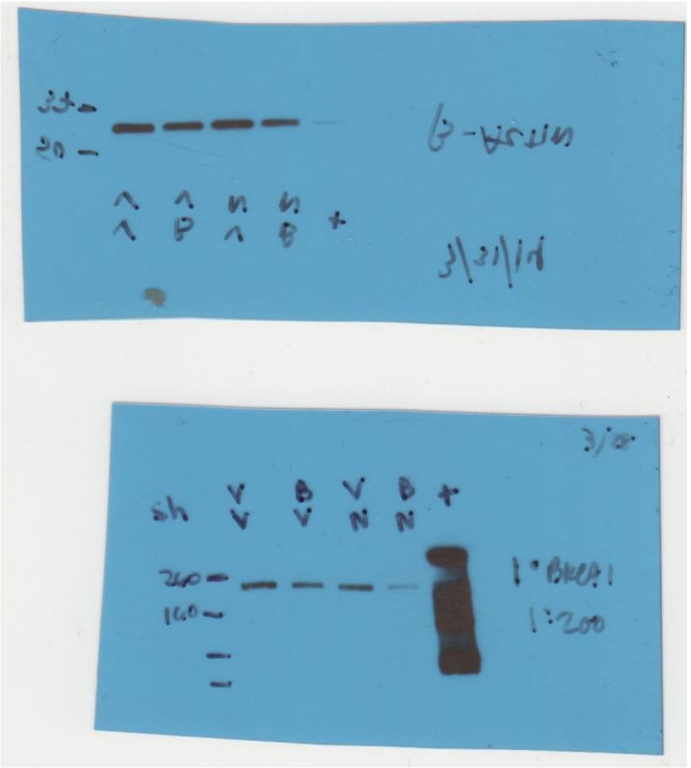

actin

BRCA1

Actin

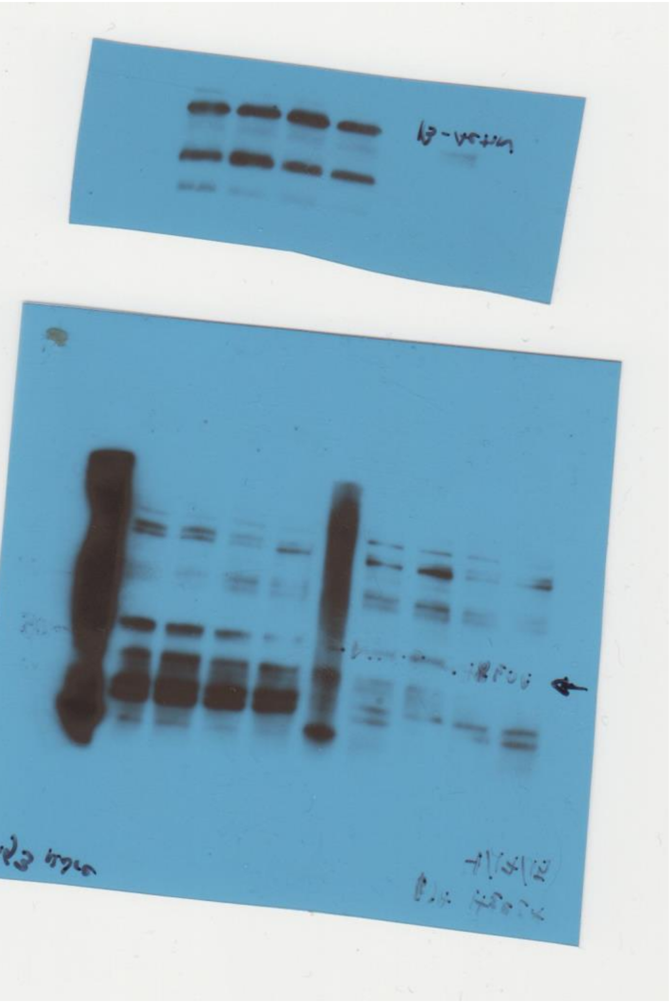

Nore1a

Figure 4

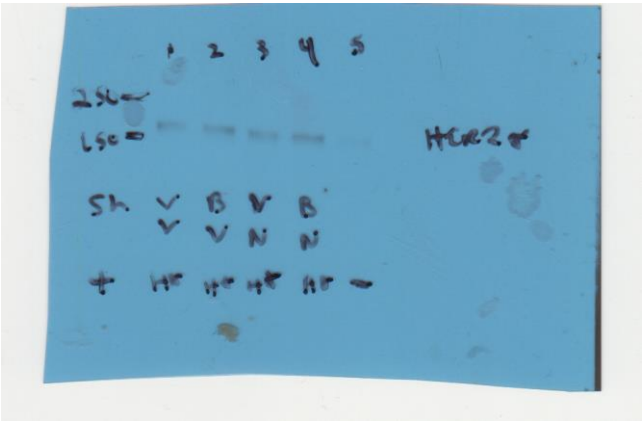

Figure 5B p21 and actin

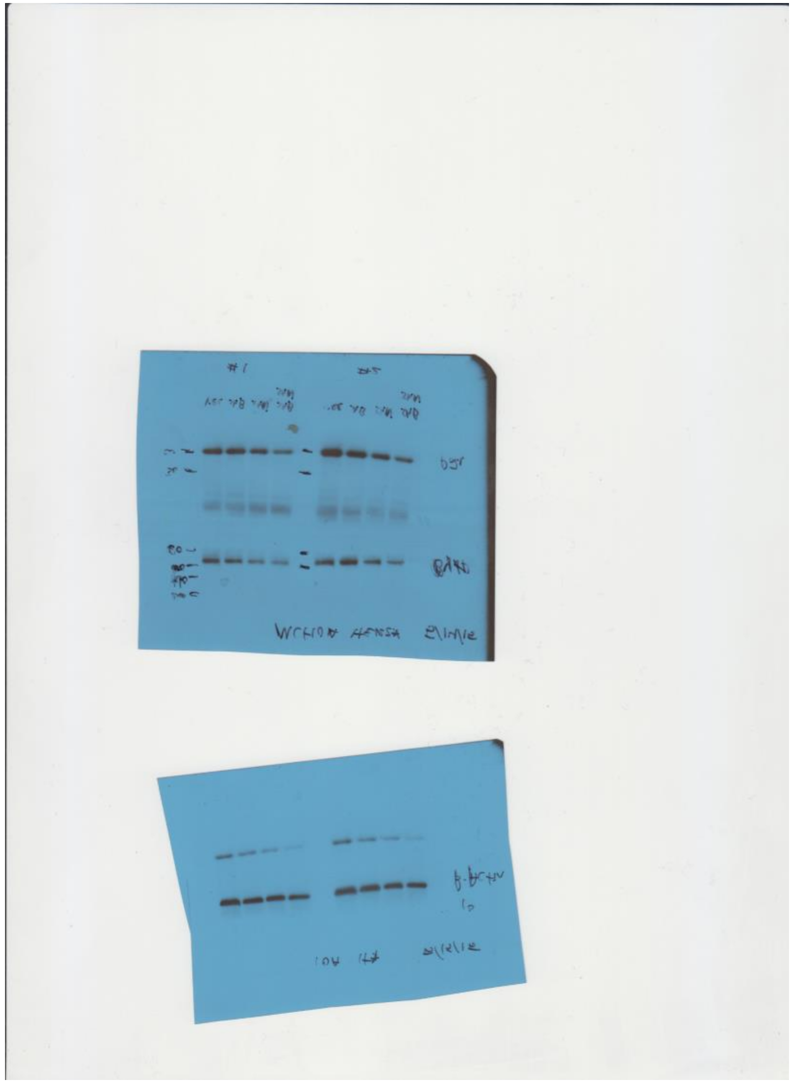

Figure 6

MCF-7 cells

BRCA1

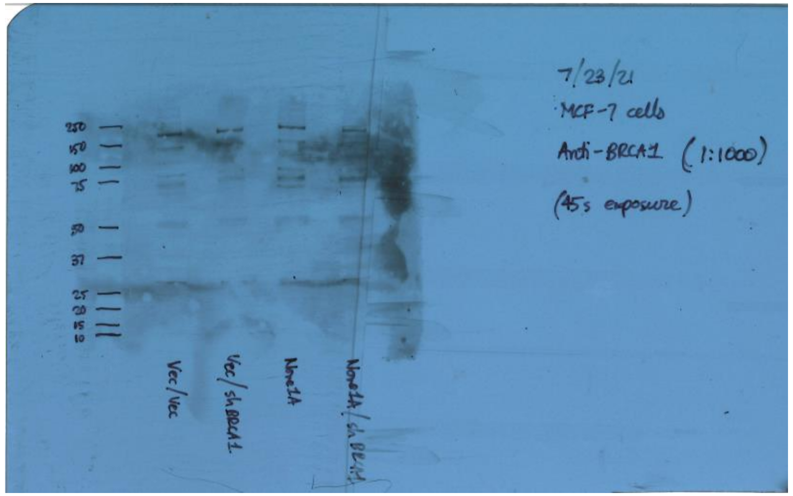

FLAG (NORE1A)

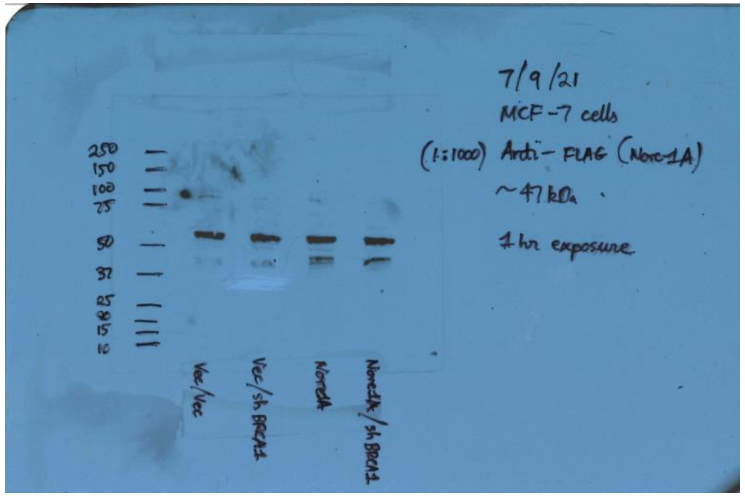

Actin

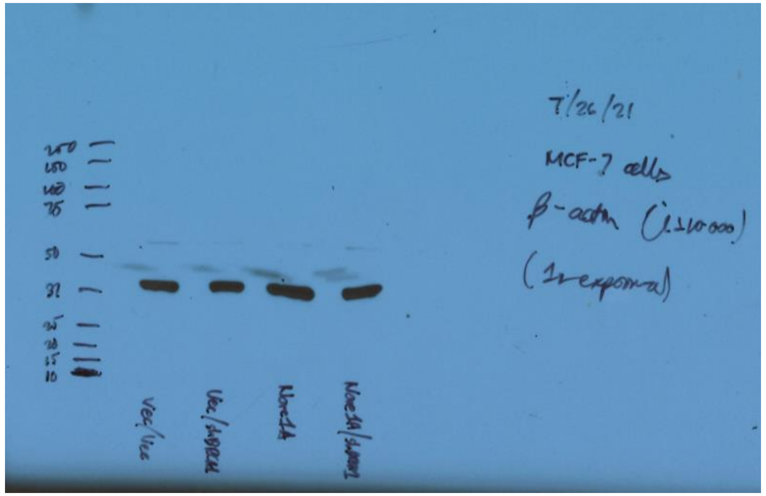

Supplement: Supplementary file 1 [file cancers-15-04133-s001.zip › cancers-2523777-supplementary.pdf]
